# Elevated Salt or Angiotensin II Levels Induce CD38+ Innate Immune Cells in the Presence of Granulocyte-Macrophage Colony Stimulating Factor

**DOI:** 10.3390/cells13151302

**Published:** 2024-08-04

**Authors:** Hannah L. Smith, Bethany L. Goodlett, Shobana Navaneethabalakrishnan, Brett M. Mitchell

**Affiliations:** Department of Medical Physiology, Texas A&M School of Medicine, Bryan, TX 77807, USA; hannahsmith@tamu.edu (H.L.S.);

**Keywords:** hypertension, macrophages, dendritic cells, CD38, granulocyte-macrophage colony stimulating factor

## Abstract

Hypertension (HTN) impacts almost half of adults, predisposing them to cardiovascular disease and renal damage. Salt-sensitive HTN (SSHTN) and angiotensin II (A2)-induced HTN (A2HTN) both involve immune system activation and renal innate immune cell infiltration. Subpopulations of activated [Cluster of differentiation 38 (CD38)] innate immune cells, such as macrophages and dendritic cells (DCs), play distinct roles in modulating renal function and blood pressure. It is unknown how these cells become CD38+ or which subtypes are pro-hypertensive. When bone marrow-derived monocytes (BMDMs) were grown in granulocyte-macrophage colony stimulating factor (GM-CSF) and treated with salt or A2, CD38+ macrophages and CD38+ DCs increased. The adoptive transfer of GM-CSF-primed BMDMs into mice with either SSHTN or A2HTN increased renal CD38+ macrophages and CD38+ DCs. Flow cytometry revealed increased renal M1 macrophages and type-2 conventional DCs (cDC2s), along with their CD38+ counterparts, in mice with either SSHTN or A2HTN. These results were replicable in vitro. Either salt or A2 treatment of GM-CSF-primed BMDMs significantly increased bone marrow-derived (BMD)-M1 macrophages, CD38+ BMD-M1 macrophages, BMD-cDC2s, and CD38+ BMD-cDC2s. Overall, these data suggest that GM-CSF is necessary for the salt or A2 induction of CD38+ innate immune cells, and that CD38 distinguishes pro-hypertensive immune cells. Further investigation of CD38+ M1 macrophages and CD38+ cDC2s could provide new therapeutic targets for both SSHTN and A2HTN.

## 1. Introduction

Hypertension (HTN) stands as the foremost contributor to cardiovascular diseases and premature mortality [1]. The NCD Risk Factor Collaboration determined that the amount of women and men aged 30–79 with HTN has reached 1.28 billion, a worldwide doubling from 1990 to 2019 [2,3]. HTN is especially problematic in the aging population, as 70% of adults 70 years or older have high blood pressure [2,3]. Salt-sensitive hypertension (SSHTN) accounts for 50% of all HTN diagnoses, placing these individuals at a three-fold higher risk of developing coronary artery disease, stroke, or chronic kidney disease [4]. Systemic or intrarenal overproduction of the vasoconstrictor hormone angiotensin II (A2) can lead to A2-induced HTN (A2HTN), sodium reabsorption, the production of aldosterone, and the release of vasopressin [5,6]. While treatments exist for these types of HTN, challenges arise in controlling blood pressure due to poor treatment compliance, poor dietary choices, and low levels of physical activity. To further complicate treatment, many affected individuals do not know they have HTN. As many anti-HTN medications focus on correcting blood pressure levels only, it is important to investigate the underlying immunological mechanisms of HTN as an area of therapeutic interest. Targeting pro-hypertensive immune cells may offer a promising approach to alleviate HTN. However, this would require furthering the understanding of how these immune cells become activated in HTN, as well as determining which immune cell phenotypes are pro-hypertensive and common in both SSHTN and A2HTN. 

A multitude of groups have reported increased accumulation of activated immune cells in the kidneys of mice with SSHTN and A2HTN [7,8,9,10,11,12,13,14,15]. Innate immune cells, such as macrophages and dendritic cells (DCs), are activated early during the development of HTN and infiltrate the kidneys in response to chemokines and other signaling factors, supporting a pro-inflammatory renal environment in HTN [16,17,18,19]. Pro-inflammatory macrophages can disrupt the vasculature by producing reactive oxygen species (ROS), leading to oxidative stress and impaired sodium excretion [20]. Similar to macrophages, DC ROS production can be stimulated by A2, leading to the formation of isoketals, which accumulate in DCs [18,21]. Isoketals are reactive to lysine residues of proteins in injured kidney tissue, which in turn form isoketal protein adducts that are perceived as non-self, further promoting the release of pro-inflammatory cytokines which in turn increase the production of activated immune cells [18,21]. 

Cluster of differentiation 38 (CD38) has been implicated as a pro-inflammatory, multifunctional ecto-enzymatic protein. CD38 expression induces pro-inflammatory macrophage and DC phenotypes [22,23,24]. Therefore, it can be used as an activation marker expressed on cells of the innate and adaptive immune systems [24]. HTN has been characterized as a chronic inflammatory condition and it has been reported that CD38 increases in HTN due to an array of inflammatory mediators [25,26]. HTN predominantly affects the aging population, and aging is associated with nicotinamide adenine dinucleotide (NAD) deficiency [27]. CD38 has been linked to the aging process, as it has binding sites for NAD [28]. When CD38 knockout mice are infused with A2, blood pressure is significantly attenuated [26,29]. Mice with A2HTN treated with two different CD38 inhibitors experienced a significant decrease in blood pressure [26]. Mice infused with A2 have increased levels of CD38 in their aortic tissue [29]. Taken together, these results suggest a pro-hypertensive nature of CD38, but whether increased renal CD38+ innate immune cells are associated with SSHTN and A2HTN is unknown. 

In this study, we hypothesized that the hypertensive stimuli salt and A2 similarly induce bone marrow-derived monocytes (BMDMs) into CD38+ innate immune cells in the presence of granulocyte-macrophage colony stimulating factor (GM-CSF), resulting in increased CD38+ bone marrow-derived (BMD) macrophages (BMD-Macs) and CD38+ bone marrow-derived DCs (BMD-DCs). We also determined whether BMDMs primed with GM-CSF, labeled, and adoptively transferred into mice with SSHTN or A2HTN would turn into CD38+ innate immune cells and infiltrate the kidney. We also identified subsets of renal CD38+ innate immune cells commonly expressed in mice with SSHTN or A2HTN. We then examined the ability of salt and A2 on BMDMs in vitro to induce the CD38+ innate immune cell subsets commonly expressed in kidneys from mice with SSHTN and A2HTN. 

## 2. Materials and Methods

### 2.1. Salt-Sensitive Hypertension and Angiotensin II-Induced Hypertension Mouse Models 

Wild-type C57BL/6J mice were purchased from Jackson Laboratories (Bar Harbor, ME) at age 8–10 weeks and acclimatized to our facility for 2 weeks. To induce salt-sensitive hypertensin (SSHTN), male mice were given nitro-l-arginine methyl ester hydrochloride (L-NAME.; 0.5 mg/mL.; Sigma, St. Louis, MO, USA) in their drinking water for 2 weeks, followed by a 2-week washout period, after which they received a 4% salt diet (Teklad Envigo, Huntingdon, United Kingdom) for 3 weeks [30,31]. Mice in the control group received L-NAME in their drinking water for 2 weeks, followed by a 2-week washout period, and standard chow through the duration of the 7-week model. All water and diets were provided ad libitum. 

To induce angiotensin II-induced hypertension (A2HTN), male mice 10–14 weeks of age were surgically implanted under inhaled isoflurane (1.5–5%) anesthesia with osmotic mini pumps (Alzet, model 1004, Cupertino, CA, USA) containing A2 (1000 ng/kg/min; Bachem, Torrance, CA, USA), followed by topical lidocaine. At the conclusion of the 3-week model, mice were euthanized by exsanguination and tissue perfusion under deep isoflurane anesthesia prior to tissue collection. 

Both of these models were associated with increased systolic blood pressure, pro-inflammatory gene expression, immune cell infiltration, and fractional excretion of sodium [31,32,33,34]. 

### 2.2. In Vitro Cell Culture of Bone Marrow-Derived Monocytes, Bone Marrow-Derived Macrophages, and Bone Marrow-Derived Dendritic Cells 

Mice were euthanized by exsanguination under 5% inhaled isoflurane anesthesia, with death confirmed by cervical dislocation. The whole mouse was doused in 70% ethanol and taken into the laminar flow hood for sterile harvest of leg bones. After removal of the fur, the back legs (femur and tibia) of the mouse were separated from the body. The remaining tissue, muscle, and feet were removed from the femur and tibia. Then, the bones were separated at the knee joint, sprayed with ethanol, and rinsed with sterile Dulbecco’s phosphate-buffered saline (DPBS.; Thermo Fisher Scientific, Waltham, MA, USA). Using scissors, one end of each bone was cut carefully. Each bone was placed into a small centrifuge tube with a hole in the bottom [35]. The small centrifuge tube was inserted into a larger 1.5 mL centrifuge tube that contained 100 µL of the appropriate cell culture media. The set of tubes containing the bones was kept on ice until the bone harvest was complete. The set of tubes was spun in a 4 °C microcentrifuge for no more than 2 min at 5000 rpm to isolate the bone marrow from the bones. From this point forward, all steps were performed on ice and were kept sterile. The supernatant was aspirated, and bone marrow pellets were resuspended and combined. The cells were filtered through a 70 μm filter into a conical tube and spun at 400 × g for 7 min with the brake off. Supernatant was aspirated and the pellet was resuspended in 3 mL of ACK Lysis Buffer (Thermo Fisher Scientific) and incubated on ice for 2 min. The reaction was halted with the addition of the appropriate cell culture media. The conical tube was spun at 400 × g for 5 min at 4 °C. The media was aspirated, and the pellet resuspended. The cells were filtered once more through a 40 μm filter into a new 50 mL conical tube. Cell total and viability were determined with a VI-Cell XR Cell Viability Analyzer (Beckman Coulter, Brea, CA, USA) and cells were plated in 6-well plates at 1.5 × 10^6^–2.5 × 10^6^. 

On day 0, cells were plated in 3 mL of media with 25 ng/mL of macrophage-colony stimulating factor (M-CSF.; R&D Systems, Minneapolis, MN) or granulocyte-macrophage colony stimulating factor (GM-CSF) (R&D Systems) and incubated at 37 °C with 5% CO^2^ [36,37]. For BMD-Macs, complete media changes took place on days 3 and 6, with the additional treatment of hypertensive stimuli on day 6 (described below). For BMD-DCs, complete media changes occurred on days 2, 4, and 6, with treatment of hypertensive stimuli on day 6. On day 6, in addition to the media change, BMD-Macs and BMD-DCs were each treated with either salt (180 mm; Sigma) or A2 (10 µM.; Bachem) for 24 h. Cells were harvested on day 7 after the 24 h treatment point. 

On day 7, TrypLE (Thermo Fisher Scientific) was warmed to 37 °C. Conditioned cell media was collected in 15 mL tubes. To collect any non-adherent cells, these tubes were spun down at 400 × g for 5 min at 4 °C. During this centrifugation, each well of the plate was treated with 1 mL of TrypLE. The plates were incubated at 37 °C for no more than 5 min. After the incubation, a cell scraper was used to dislodge adherent cells from the plate. To stop the reaction, 3 mL of the appropriate cell media was added to each well. At this point, the tubes with conditioned media were collected and the conditioned media was decanted into long-term storage conical tubes for later use, leaving cell pellets in the original tubes. The harvested cells from the plate were added into their respective 15 mL conical tubes with the cell pellets. After centrifugation, the media was aspirated, and the cells were rinsed with DPBS before proceeding to downstream analyses. 

To differentiate BMD-Macs and BMD-DCs from bone marrow-derived monocytes (BMDMs), specific media was used for each plating cycle. For BMD-Macs, cells were grown in DMEM high-glucose media (Thermo Fisher Scientific) with 10% FBS (heat-inactivated; Sigma) and 1% penicillin/streptomycin (Thermo Fisher Scientific) [38]. For BMD-DCs, the media used was RPMI 1640 (Thermo Fisher Scientific) with 10% FBS, 1 mM HEPES (Cytiva Life Sciences, Marlborough, MA, USA), 1% penicillin/streptomycin, and 50 µM b-mercaptoethanol (Sigma) [39].

### 2.3. Adoptive Transfer of Granulocyte-Macrophage Colony Stimulating Factor-Primed Bone Marrow-Derived Monocytes

On day 7 of the BMDM protocol, untreated BMDMs grown in GM-CSF were harvested and stained with CellTracker™ Deep Red dye (Thermo Fisher Scientific) to track immune cell movement and allow for the identification of cell phenotypes via flow cytometry. The dyes were concentrated according to the manufacturer’s working dye solution recommendations. In total, 1 × 10^6^ live cells were isolated from the harvested cells by cell count [40]. The cells were incubated with CellTracker™ Deep Red dye for 45 min at 37 °C. After the incubation, the cells were centrifuged at 400× *g* for 3 min at 4 °C, and the working dye solution was removed. The cell pellet was then resuspended with 200 µL of DPBS. Mice with either SSHTN or A2HTN as described above were injected intraperitoneally with the cells using an insulin needle. Twelve hours later, the mice were euthanized, and the kidneys were isolated for flow cytometry. 

### 2.4. Flow Cytometry

**Figure 1:**
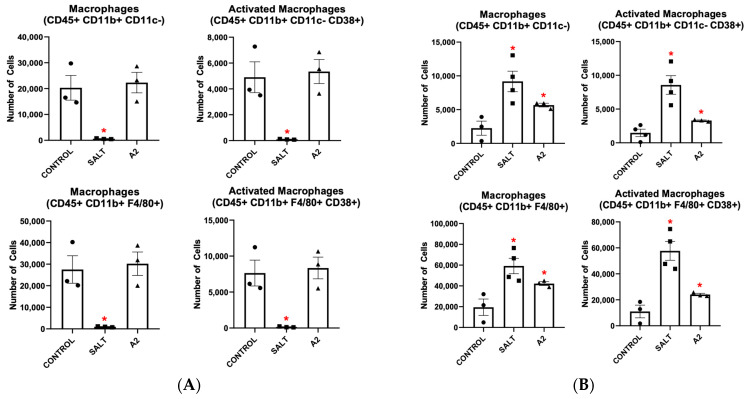
Hypertensive stimuli increase bone marrow-derived macrophages and CD38+ bone marrow-derived macrophages when bone marrow-derived monocytes are grown in the presence of granulocyte-macrophage colony stimulating factor but not macrophage colony stimulating factor. Macrophage populations resulting from salt or A2 treatment of BMDMs grown in macrophage media in the presence of (**A**) M-CSF or (**B**) GM-CSF. Data are presented as individual values and means ± SEM with *n* = 3 for control, *n* = 3 for salt, and *n* = 3 for A2. Data were analyzed with unpaired Student’s *t*-test, * *p* < 0.05 vs. control.

**Figure 2:**
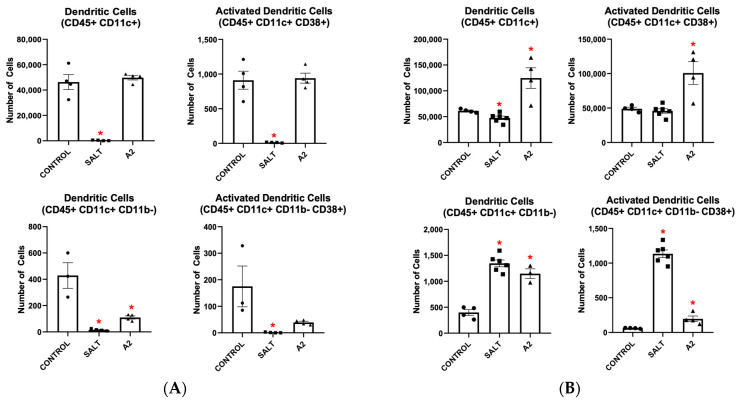
Hypertensive stimuli increase bone marrow-derived dendritic cells and CD38+ bone marrow-derived dendritic cells only when bone marrow-derived monocytes are grown in the presence of granulocyte-macrophage colony stimulating factor but not macrophage colony stimulating factor. DC populations resulting from salt and A2 treatment of BMDMs grown in DC media in the presence of (**A**) M-CSF or (**B**) GM-CSF. Data are presented as individual values and means ± SEM with *n* = 3–4 for control, *n* = 4–6 for salt, and *n* = 3–4 for A2. Data were analyzed using unpaired Student’s *t*-test, * *p* < 0.05 vs. control.

**Figure 3:**
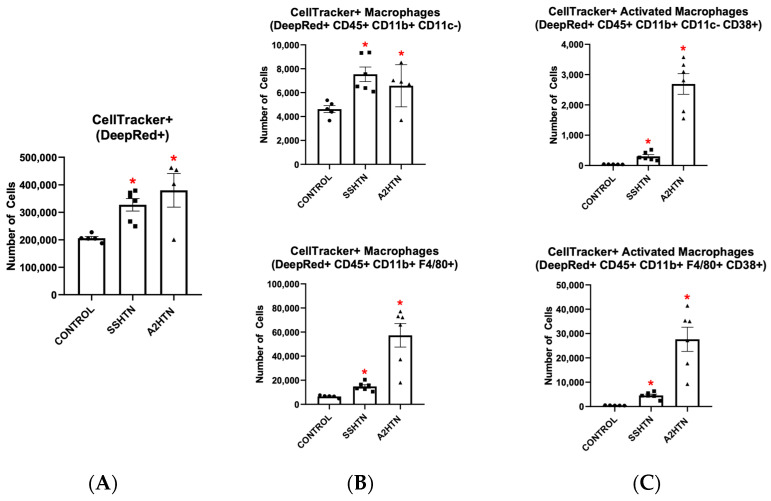
Adoptive transfer of granulocyte-macrophage colony stimulating factor-primed bone marrow-derived monocytes into mice with salt-sensitive hypertension and angiotensin II-induced hypertension led to increased macrophages and CD38+ macrophages in the kidneys. Populations of renal (**A**) CellTracker+ cells, (**B**) CellTracker+ macrophages, and (**C**) CellTracker+ CD38+ macrophages 12 h after adoptive transfer of BMDMs into mice with either SSHTN or A2HTN. Data are presented as individual values and means ± SEM with *n* = 5 for control, *n* = 6 for SSHTN, and *n* = 4–6 for A2HTN. Data were analyzed using unpaired Student’s *t*-test, * *p* < 0.05 vs. control.

**Figure 4:**
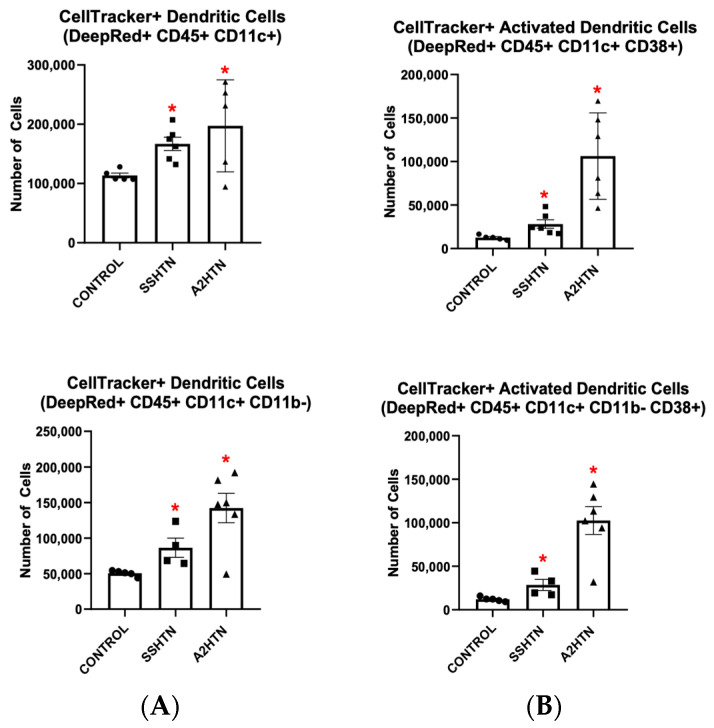
Adoptive transfer of granulocyte-macrophage colony stimulating factor-primed bone marrow-derived monocytes into mice with salt-sensitive hypertension and angiotensin II-induced hypertension led to increased dendritic cells and CD38+ dendritic cells in the kidneys. Populations of renal (**A**) CellTracker+ macrophages and (**B**) CellTracker+ CD38+ macrophages 12 h after adoptive transfer of BMDMs into mice with either SSHTN or A2HTN. Data are presented as individual values and means ± SEM with *n* = 5 for control, *n* = 4–6 for SSHTN, and *n* = 5–6 for A2HTN. Data were analyzed using unpaired Student’s *t*-test, * *p* < 0.05 vs. control.

**Figure 5:**
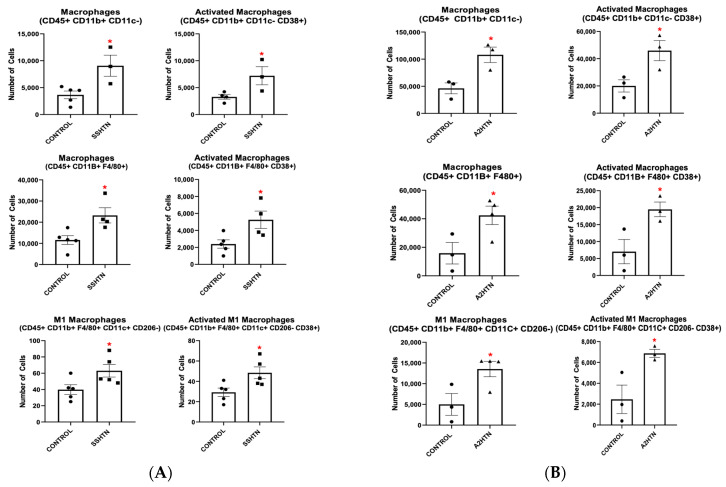
Renal macrophages and M1 macrophages are increased and activated in salt-sensitive hypertension and angiotensin II-induced hypertension. Macrophage populations in the kidneys of mice with (**A**) SSHTN and (**B**) A2HTN. Data are presented as individual values and means ± SEM with *n* = 3–5 for control, *n* = 3–5 for SSHTN, and *n* = 3–4 for A2HTN. Data were analyzed using unpaired Student’s *t*-test, * *p* < 0.05 vs. control.

**Figure 6:**
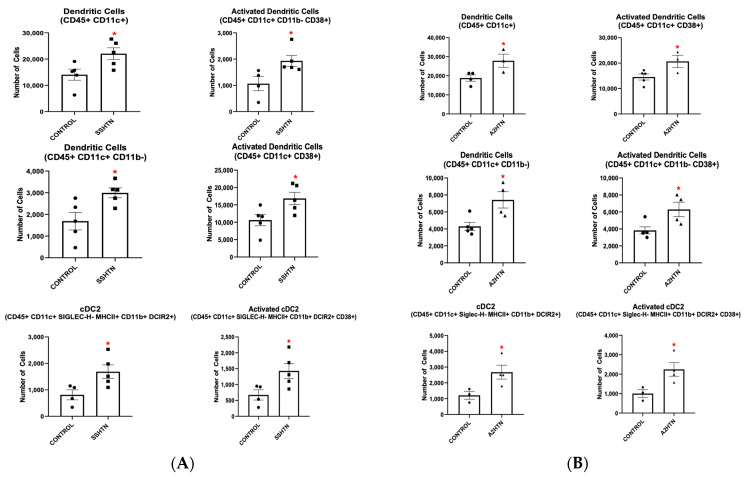
Renal dendritic cells and type-2 conventional dendritic cells are increased and activated in salt-sensitive hypertension and angiotensin II-induced hypertension. Renal DC populations in the kidneys of mice with (**A**) SSHTN and (**B**) A2HTN. Data are presented as individual values and means ± SEM with *n* = 3–5 for control, *n* = 5 for SSHTN, and *n* = 3–4 for A2HTN. Data were analyzed using unpaired Student’s *t*-test, * *p* < 0.05 vs. control.

**Figure 7:**
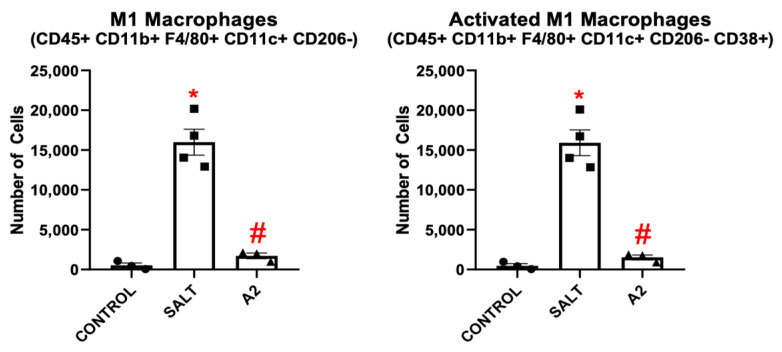
Hypertensive stimuli increase bone marrow-derived M1 macrophages and CD38+ bone marrow-derived M1 macrophages in vitro. BMD-M1 and CD38+ BMD-M1 macrophage populations following 24 h of treatment with salt or A2. Data are presented as individual values and means ± SEM with *n* = 3 for control, *n* = 4 for salt, and *n* = 3 for A2. Data were analyzed using unpaired Student’s *t*-test, * *p* < 0.05 and # *p* < 0.1 vs. control.

**Figure 8:**
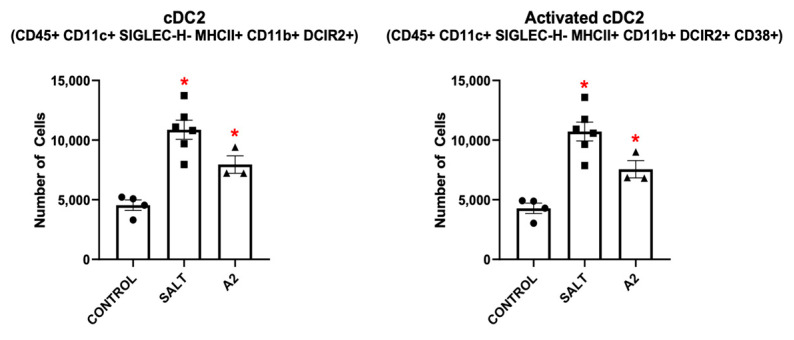
Hypertensive stimuli increase bone marrow-derived type-2 conventional dendritic cells and CD38+ bone marrow-derived type-2 conventional dendritic cells in vitro. cDC2 populations following 24 h of treatment with salt or A2. Data are presented as individual values and means ± SEM with *n* = 4 for control, *n* = 6 for salt, and *n* = 3 for A2. Data were analyzed using unpaired Student’s *t*-test, * *p* < 0.05 vs. control.

Kidneys were decapsulated, minced thoroughly, and added to digestion buffer containing collagenase D (2.5 mg/mL.; Sigma) and dispase II (1 mg/mL.; Sigma). Kidneys were digested into single-cell suspensions by incubating samples at 37 °C for 30 min with constant disruption using a gentleMACS Octo Dissociator with heaters (Miltenyi Biotec, Bergisch Gladbach, Germany). Following digestion, single cell suspensions of kidneys were passed through sterile 100 μm and 40 μm strainers and rinsed with DPBS. Red blood cells were lysed using ACK lysing buffer and samples were rinsed twice with DPBS prior to plating. The isolated cells were plated into 96-well conical plates and incubated with either Ghost Dye Red 710 (Tonbo Biosciences, San Diego, CA, USA) or a Zombie UV Fixable Viability Kit (BioLegend, Inc., San Diego, CA, USA), depending on the antibody panel and cytometer used, for 30 min on ice. After two DPBS washes, cells were resuspended in 0.1% FBS solution and incubated with an anti-mouse CD16/CD32 antibody (BD Biosciences, San Jose, CA, USA) for 10 min on ice to block non-specific Fc binding. Next, cells were stained for 30 min using fluorescent-conjugated antibodies against the necessary antibodies for either the Fortessa, Cytek, or Adoptive Transfer flow panels (Supplementary Figure S1a−c) at a 1:100 dilution for kidneys and 1:200 for cultured cells. All cells were washed with DPBS, resuspended in 0.1% FBS solution, and passed through sterile 35 μm strainers prior to flow cytometric analysis. Data were acquired with a BD LSR Fortessa X−20 flow cytometer with FACS DIVA software v9.0 (BD Biosciences) for Figure 1 and Figure 2. Data were acquired with a Cytek Aurora 5L (Cytek Biosciences, Fremont, CA) for Figure 3, Figure 4, Figure 5, Figure 6, Figure 7 and Figure 8. Cell populations were analyzed using FlowJo v10.8 (FlowJo, LLC, Ashland, OR, USA). Results are expressed as number of cells. Gating strategies for all samples were justified by referencing unstained specimens, compensation controls, and a DC flow cytometric methods paper [41] (Supplementary Figure S2a−c). 

In vitro immune cells analyzed with flow cytometry were harvested as described in Section 2.2, plated in 96-well conical plates, and rinsed with DPBS twice before proceeding with the staining steps described above. 

### 2.5. Statistical Analysis 

Statistical analyses were conducted with GraphPad Prism version 8.4.3 (GraphPad Software, Inc., Boston, MA, USA). Differences between control vs. SSHTN, control vs. A2HTN, control vs. salt treatment, and control vs. A2 treatment were evaluated using two-tailed unpaired Student’s *t*-tests. Results are depicted in bar graphs with dot plots showing mean ± SEM. Statistical significance (*) is defined at *p* < 0.05 and trends (#) toward significance are defined at *p* < 0.1.

## 3. Results

### 3.1. Salt and Angiotensin II Induce CD38+ Innate Immune Cells from Control Bone Marrow-Derived Monocytes in the Presence of Granulocyte-Macrophage Colony Stimulating Factor but Not Macrophage Colony Stimulating Factor

To explore how macrophages become activated (CD38+) when treated with hypertensive stimuli, isolated BMDMs from mice were cultured in macrophage media for 7 days. As shown in Figure 1A, BMDMs grown with the traditional macrophage growth factor M-CSF exhibited a dampened response when treated with either salt or A2. Fewer cells became BMD-Macs (CD45+ CD11b+ CD11c- or CD45+ CD11b+ F4/80+) and fewer BMD-Macs became CD38+ (CD45+ CD11b+ CD11c- CD38+ or CD45+ CD11b+ F4/80+ CD38+) in response to salt or A2 treatment. 

Next, we investigated the effects of GM-CSF in the macrophage growth medium in place of M-CSF. As shown in Figure 1B, BMDMs grown with GM-CSF exhibited a significantly enhanced response when treated with hypertensive stimuli. More BMDMs became BMD-Macs and more of those BMD-Macs became CD38+ following either salt or A2 treatment. 

To further explore how innate immune cells become activated in HTN, we isolated BMDMs from mice and cultured them in DC media for 7 days with either M-CSF or GM-CSF. When BMD-DCs were grown with M-CSF and treated with salt, all surveyed BMD-DC populations, including CD38+ BMD-DCs, decreased (Figure 2A). When these cells were treated with A2, BMD-DC populations remained largely unchanged or decreased (Figure 2A). However, when grown with GM-CSF, more CD45+ CD11c+ and CD45+ CD11c+ CD11b− BMD-DCs differentiated in response to salt or A2 treatment (Figure 2B). Additionally, more BMD-DCs became CD38+ when challenged with salt or A2, as seen in Figure 2B. Collectively, these findings demonstrate that GM-CSF acts as a primer for BMDMs to differentiate into an activated CD38+ pro-hypertensive macrophage and DC phenotype in response to either salt or A2.

### 3.2. Adoptive Transfer of Granulocyte-Macrophage Colony Stimulating Factor-Primed and -Labeled Bone Marrow-Derived Monocytes into Mice with Salt-Sensitive or Angiotensin II-Induced Hypertension Increases Renal CD38+ Innate Immune Cells

To further examine the role of GM-CSF in HTN, 1 × 10^6^ BMDMs were adoptively transferred into mice with either SSHTN or A2HTN. Analysis of flow cytometry data revealed that significantly more of the adoptive transferred cells trafficked to the kidneys of SSHTN or A2HTN mice compared to the control normotensive mice (Figure 3A). In both hypertensive groups, significantly more of the adoptive transferred cells turned into CD45+ CD11b+ CD11c- and CD45+ CD11b+ F4/80+ macrophages (Figure 3B). Further, significantly more of the adoptive transferred cells became CD38+ macrophages in both hypertensive models (Figure 3C). 

Flow cytometric analysis revealed similar results when renal DC populations were surveyed. Of the adoptive transferred cells that made it into either SSHTN or A2HTN kidneys, CD45+ CD11c+ and CD45+ CD11c+ CD11b− DCs, as well as their CD38+ phenotypes, were increased significantly, as seen in Figure 4A,B. Taken together, these findings demonstrate the ability of GM-CSF-primed innate immune cells to traffic to the kidneys in SSHTN and A2HTN and become CD38+ under hypertensive conditions. 

### 3.3. Renal Subsets of CD38+ Innate Immune Cells Are Increased in Mice with Salt-Sensitive Hypertension and Angiotensin II-Induced Hypertension

Next, we sought to determine specific renal macrophage populations of interest by flow cytometry. As shown in Figure 5A, CD45+ CD11b+ CD11c- and CD45+ CD11b+ F4/80+ macrophages and their corresponding CD38+ activated counterparts were all significantly increased in the kidneys of SSHTN mice. Renal classical M1 macrophages, as well as their CD38+ equivalents, were also increased significantly in SSHTN (Figure 5A). To validate the use of CD38 as an activation marker, the same cell types were analyzed for expression of CD68, a traditional activation marker for macrophages, and activated CD45+ CD11b+ CD11c- macrophages and M1 macrophages were found to be significantly increased (Supplementary Figure S3a). 

To further investigate renal macrophages and their CD38+ counterparts in HTN, renal macrophage populations were also assessed in the A2HTN model. Similar to what was seen in SSHTN, CD45+ CD11b+ CD11c- and CD45+ CD11b+ F4/80+ macrophages were increased significantly in the kidneys of A2HTN mice, along with their corresponding CD38+ activation states. M1 and CD38+ M1 macrophages were also increased significantly in A2HTN kidneys. These results highlight the involvement of multiple macrophage phenotypes in SSHTN and A2HTN, as well as the importance of CD38 as a pro-hypertensive activated macrophage marker. 

Next, we identified DC populations of interest in kidneys from mice with either SSHTN or A2HTN via flow cytometry. CD45+ CD11c+ and CD45+ CD11c+ CD38+ DCs, as well as CD45+ CD11c+ CD11b− and CD45+ CD11c+ CD11b− CD38+ DCs, were increased significantly in SSHTN kidneys (Figure 6A). Renal type-2 conventional DCs (cDC2s) and activated CD38+ cDC2s were also increased significantly in SSHTN (Figure 6A). Similar results were found for the expression of CD86, a traditional DC activation marker (Supplementary Figure S3b). 

Mice with A2HTN had increased renal CD45+ CD11c+ and CD45+ CD11c+ CD11b− DCs, as well as increases in their CD38+ activated states (Figure 6B). Similar to the SSHTN group, the A2HTN group had increased cDC2s and CD38+ cDC2s (Figure 6B). Analysis for CD86 expression showed significant increases in both CD86+ DCs and CD86+ cDC2s, further validating CD38 as an activation marker for DCs and cDC2s (Supplementary Figure S3c). These results support the notion that multiple DC phenotypes are increased similarly in SSHTN and A2HTN and underscore the importance of CD38+ as a pro-hypertensive activated DC marker.

As SSHTN and A2HTN are different models of HTN that have different mechanisms, some changes in innate immune cell populations varied between the models. Renal M2 macrophages were increased significantly in A2HTN, but not in SSHTN (Supplementary Figure S4a). Conversely, renal monocyte-derived DCs (moDCs) were increased significantly in SSHTN, but not in A2HTN (Supplementary Figure S4b). Renal type-1 conventional DCs (cDC1s) were increased in both SSHTN and A2HTN; however, these results were not fully replicated in vitro (Supplementary Figure S4c). 

We also investigated a potentially refined gating strategy for cDC2s. Supplementary Figure S4d presents an alternate strategy which excludes CD11b, a traditional macrophage marker, and focuses on DCIR2. The results are similar to those presented in Figure 6A,B and Figure 8, which had CD11b in their gating strategies. Thus, DCIR2 may be a marker for cDC2s that is specific enough to allow for the exclusion of CD11b, which could prevent confusion regarding this unique DC subset.

Though multiple subsets of CD38+ innate immune cells were identified in either SSHTN or A2HTN, renal CD38+ M1 macrophages and CD38+ cDC2s were increased similarly in both models. These data highlight these innate immune cells of interest for further analysis of their role in HTN. 

### 3.4. Salt and Angiotensin II Induce CD38+ M1 Macrophages and CD38+ Type-2 Conventional Dendritic Cells

To further demonstrate the importance of macrophage phenotypes in HTN, they were replicated in vitro by priming BMDMs with GM-CSF and treating with hypertensive stimuli. When BMDMs were treated with salt, there was a significant increase in BMD-Macs and CD38+ BMD-Macs (Figure 1B), as well as bone marrow-derived M1 macrophages (BMD-M1s) and CD38+ BMD-M1s (Figure 7). To further validate CD38 as an activation marker, CD68 expression was also measured. CD45+ CD11b+ CD11c- CD68+ BMD-Macs were increased significantly when BMDMs were treated with either salt or A2 (Supplementary Figure S5a). In addition, CD68+ BMD-M1s were increased significantly when BMDMs were treated with salt (Supplementary Figure S5a). When BMDMs were treated with A2, BMD-Macs and CD38+ BMD-Macs increased significantly and BMD-M1s and CD38+ BMD-M1s trended towards significance (Figure 1B and Figure 7). A2 treatment skewed more BMDMs toward a BMD-M2 phenotype, similar to what is seen in A2HTN, whereas salt treatment did not significantly impact M2 populations (Supplementary Figure S4a). 

BMD-DCs and CD38+ BMD-DCs, as well as bone marrow-derived cDC2s (BMD-cDC2) and CD38+ BMD-cDC2s, were also induced in vitro when BMDMs were treated with either salt or A2 in the presence of GM-CSF (Figure 2B and Figure 8). Once again, we tested CD38 for its validity as an activation marker and found that, when measuring CD86 expression, activated BMD-DCs and BMD-cDC2s were still increased significantly in vitro after treatment with salt or A2 (Supplementary Figure S5b). BMD-moDCs were increased after treatment with salt or A2 (Supplementary Figure S4b). BMD-cDC1s were increased only when treated with A2 (Supplementary Figure S4c). Similar to in vivo results, BMD-cDC2s gated without CD11b along with their CD38+ counterpart were also increased with either salt or A2 treatment (Supplementary Figure S4d). 

To shed light on the replicability of the immune cell phenotypes found in vivo, BMDMs were cultured with GM-CSF and treated with salt or A2. This resulted in the reproduction of CD38+ M1 macrophages as well as CD38+ cDC2s. This sets a foundation for common innate immune cell subsets that may be involved in HTN in general and brings forth the opportunity for them to be studied more effectively in vivo and in vitro.

## 4. Discussion

CD38+ innate immune cells are a multifaceted group of cells associated with inflammation and HTN. In this study, we determined that GM-CSF is necessary for BMDMs to replicate the immune cell phenotypes found in vivo in response to treatment with salt or A2. Adoptive transfer of GM-CSF-primed BMDMs resulted in more macrophages and DCs trafficking to hypertensive kidneys, as well as more of these cells differentiating into a CD38+ activated phenotype. When analyzing SSHTN and A2HTN renal innate immune cell populations, both models demonstrated increases in CD38+ macrophages and CD38+ DCs. Other innate immune cells, such as M2 macrophages, moDCs, and cDC1s, shed light on specific innate immune cell differences across HTN models. 

Notably, M1 macrophages, CD38+ M1 macrophages, cDC2s, and CD38+ cDC2s were the only immune cell populations that were increased similarly in both SSHTN and A2HTN kidneys and were able to be replicated in vitro after treatment with either salt or A2. Specifically, these phenotypes were replicated in vitro once GM-CSF was introduced to the culture medium in replacement of M-CSF. GM-CSF is known to be elevated in inflammatory and autoimmune diseases [42]. GM-CSF is also elevated in the plasma and serum of humans with HTN [43,44,45]. GM-CSF binding activates multiple pathways involved in HTN, including signal transducer and activator of transcription 5 (STAT5) which initiates Janus Kinase (JAK) 2 signaling, and the Nuclear Factor kappa-light-chain-enhancer of activated B cell pathways (NFκB) [46,47,48]. The JAK/STAT pathway is involved in the upregulation of A2 production from the renin–angiotensin–aldosterone system, further contributing to HTN [49]. The NFκB pathway has previously been reported to reduce blood pressure when blocked in models of HTN [50]. When considered with the data from the current study, this information potentiates future studies to identify a mechanistic link between GM-CSF, salt, A2, and CD38+ immune cell phenotypes.

Previous adoptive transfer studies have reported that macrophages traffic to the kidneys and can induce renal damage in inflammatory models [51]. Barbaro et al. reported that when salt-treated DCs are adoptively transferred into mice receiving low-dose A2, mice develop HTN [40]. This helped lead to our hypothesis that CD38+ macrophages and CD38+ DCs may play a role in both SSHTN and A2HTN. CD38 has a variety of roles and, as a cell surface receptor, it is important for immune cell activation and proliferation. M1 macrophages are able to upregulate CD38 expression more than 50 times via stimulation with lipopolysaccharide (LPS) and interferon-gamma, and we believe salt and A2 may be functioning in a similar manner to LPS [52,53]. Renal innate immune cells, all of which can be CD38+, are linked to calcium signaling and regulation, immune cell regulation, oxidative stress and inflammation, fibrosis, and tissue remodeling. Additionally, these cells play roles in pathological kidney diseases, deeming them worthy for investigation in HTN and potential therapeutic avenues. In this study, we confirm that CD38 is a reliable activation marker for macrophages, M1 macrophages, DCs, and cDC2s. We have also demonstrated that these CD38+ phenotypes are readily found in hypertensive kidneys and that more of them are produced when GM-CSF-grown BMDMs are adoptively transferred and trafficked to hypertensive kidneys. These findings support a role for CD38 in HTN. 

Qui et al. report that CD38 knockout mice have decreased blood pressure in an A2HTN model; however, they thought it was due to CD38+ endothelial cells rather than BMD immune cell derivatives [26]. Similarly, Gan et al. observed decreased blood pressure in a CD38 knockout hypertensive mouse model, although they attributed it to CD38+ vascular smooth muscle cells and not CD38+ lymphocytes [29]. Contrary to the aforementioned studies, it has been reported that CD38 knockout mice are able to develop A2HTN [54]. Innate or adaptive immune cells can become activated by a variety of methods differentiating them into CD38+ immune cells. Here, we demonstrate that BMDMs not only become CD38+ after challenge with two types of hypertensive stimuli, but also traffic to the kidneys in mice with both SSHTN and A2HTN. We also identified significant increases in CD38+ immune cells in hypertensive kidneys, showcasing the importance of the bone marrow-derived immune cells in two models of HTN. These data highlight a possible role for CD38+ innate immune cells in the pathogenesis of HTN.

M1 macrophages play a significant role in the pathogenesis of HTN. They contribute to HTN via inflammatory cytokine production, oxidative stress, vascular remodeling, and renal inflammation. Renal macrophage accumulation in HTN has been established as a pathological hallmark [55,56]. Huang et al. found that depleting macrophages in both SSHTN and A2HTN results in decreased blood pressure and attenuates renal damage [56]. It has also been established that mice lacking M-CSF have decreased macrophages and minimal blood pressure elevation in A2HTN [57]. These results not only identify a prominent role of pro-inflammatory activated macrophages in HTN, but also point toward GM-CSF, a dominant inflammatory marker, as an untapped area of interest in HTN. The roles of CD38+ M1 macrophages and GM-CSF in HTN should be further explored, as the identification of new therapeutic targets may result. 

Little is known about cDC2 cells and HTN. Srinivas et al. reported a decrease in splenic cDC2s in the two-kidney-one-clip renovascular model of HTN [58]. Notable differences exist between this research and our current study in regard to the model of HTN used, the organ the cells are obtained from, and the specificity of gating for flow cytometry. In regard to characterizing splenic cDC2s, the spleen is known to have a range of DC subsets dependent on anatomic location within the organ [59]. It is also known that splenic DCs express DCIR2, which could have further validated the cDC2 phenotypes they found in the spleen [59,60]. There is great value in using DCIR2 as an additional marker to define cDC2s, since CD11b+ DCs exclusively express DCIR2, and DCIR2 is a highly specific DC marker [60,61,62,63]. Elucidating further the role of CD38+ cDC2s is a vital target in both models of HTN presented here.

The present study provides evidence that CD38 is a marker for hypertensive immune cells in both SSHTN and A2HTN. Our data suggest that GM-CSF, in addition to salt or A2, plays a role in innate immune cell activation both in vivo and in vitro. We also identified two innate immune cell phenotypes, CD38+ M1 macrophages and CD38+ cDC2s, that are found in kidneys in both SSHTN and A2HTN. These innate immune cell phenotypes are able to be replicated and induced in vitro after treatment with salt or A2 in the presence of GM-CSF, further supporting their roles in HTN. Further characterization of these innate immune cell subsets is underway. 

## Data Availability

Data are contained within the article or Supplementary Material.

## Supplementary Materials

The following supporting information can be downloaded at: https://www.mdpi.com/article/10.3390/cells13151302/s1, Supplementary Figure S1: Antibody panels for flow cytometry; Supplementary Figure S2: Flow cytometry gating strategies for innate immune cells; Supplementary Figure S3: Activated macrophages and M1 macrophages are increased in SSHTN and DCs and cDC2s are increased in SSHTN and A2HTN.; Supplementary Figure S4: SSHTN and A2HTN in vivo and salt and A2 treatment in vitro differentially affect innate immune cell populations; Supplementary Figure S5: Treatment of BMDMs with hypertensive stimuli increases activated macrophages, M1 macrophages, DCs, and cDC2s.

## References

[1] Mills K.T., Stefanescu A., He J. (2020). The global epidemiology of hypertension. Nat. Rev. Nephrol..

[2] Schutte A.E., Jafar T.H., Poulter N.R., Damasceno A., Khan N.A., Nilsson P.M., Alsaid J., Neupane D., Kario K., Beheiry H. (2023). Addressing global disparities in blood pressure control: Perspectives of the International Society of Hypertension. Car-diovasc. Res..

[3] Collaboration N.C.D.R.F. (2021). Worldwide trends in hypertension prevalence and progress in treatment and control from 1990 to 2019: A pooled analysis of 1201 population-representative studies with 104 million participants. Lancet.

[4] Kotchen T.A., Cowley A.W., Frohlich E.D. (2013). Salt in health and disease—A delicate balance. N. Engl. J. Med..

[5] Fountain J.H., Kaur J., Lappin S.L. (2023). Physiology, Renin Angiotensin System.

[6] Moon J.Y. (2013). Recent Update of Renin-angiotensin-aldosterone System in the Pathogenesis of Hypertension. Electrolyte Blood Press..

[7] Crowley S.D., Song Y.S., Lin E.E., Griffiths R., Kim H.S., Ruiz P. (2010). Lymphocyte responses exacerbate angiotensin II-dependent hypertension. Am. J. Physiol. Regul. Integr. Comp. Physiol..

[8] Ozawa Y., Kobori H., Suzaki Y., Navar L.G. (2007). Sustained renal interstitial macrophage infiltration following chronic angiotensin II infusions. Am. J. Physiol. Renal. Physiol..

[9] Rodriguez-Iturbe B., Quiroz Y., Herrera-Acosta J., Johnson R.J., Pons H.A. (2002). The role of immune cells infiltrating the kidney in the pathogenesis of salt-sensitive hypertension. J. Hypertens. Suppl..

[10] De Miguel C., Das S., Lund H., Mattson D.L. (2010). T lymphocytes mediate hypertension and kidney damage in Dahl salt-sensitive rats. Am. J. Physiol. Regul. Integr. Comp. Physiol..

[11] De Miguel C., Lund H., Mattson D.L. (2011). High dietary protein exacerbates hypertension and renal damage in Dahl SS rats by increasing infiltrating immune cells in the kidney. Hypertension.

[12] Franco M., Tapia E., Bautista R., Pacheco U., Santamaria J., Quiroz Y., Johnson R.J., Rodriguez-Iturbe B. (2013). Impaired pressure na-triuresis resulting in salt-sensitive hypertension is caused by tubulointerstitial immune cell infiltration in the kidney. Am. J. Physiol. Renal. Physiol..

[13] Rodriguez-Iturbe B. (2010). Renal infiltration of immunocompetent cells: Cause and effect of sodium-sensitive hypertension. Clin. Exp. Nephrol..

[14] Rodriguez-Iturbe B., Franco M., Tapia E., Quiroz Y., Johnson R.J. (2012). Renal inflammation, autoimmunity and salt-sensitive hypertension. Clin. Exp. Pharmacol. Physiol..

[15] Takebayashi S. (1969). Ultrastructural studies on glomerular lesions in experimental hypertension. Acta. Pathol. Jpn..

[16] Crowley S.D., Frey C.W., Gould S.K., Griffiths R., Ruiz P., Burchette J.L., Howell D.N., Makhanova N., Yan M., Kim H.S. (2008). Stimulation of lymphocyte responses by angiotensin II promotes kidney injury in hypertension. Am. J. Physiol. Renal. Physiol..

[17] Mouton A.J., Li X., Hall M.E., Hall J.E. (2020). Obesity, Hypertension, and Cardiac Dysfunction: Novel Roles of Immunometabolism in Macrophage Activation and Inflammation. Circ. Res..

[18] Kirabo A., Fontana V., de Faria A.P., Loperena R., Galindo C.L., Wu J., Bikineyeva A.T., Dikalov S., Xiao L., Chen W. (2014). DC isoketal-modified proteins activate T cells and promote hypertension. J. Clin. Invest..

[19] McMaster W.G., Kirabo A., Madhur M.S., Harrison D.G. (2015). Inflammation, immunity, and hypertensive end-organ damage. Circ. Res..

[20] Justin Rucker A., Crowley S.D. (2017). The role of macrophages in hypertension and its complications. Pflug. Arch..

[21] Caillon A., Paradis P., Schiffrin E.L. (2019). Role of Immune cells in hypertension. Br. J. Pharmacol..

[22] Lischke T., Heesch K., Schumacher V., Schneider M., Haag F., Koch-Nolte F., Mittrucker H.W. (2013). CD38 controls the innate immune response against Listeria monocytogenes. Infect. Immun..

[23] Fedele G., Frasca L., Palazzo R., Ferrero E., Malavasi F., Ausiello C.M. (2004). CD38 is expressed on human mature monocyte-derived dendritic cells and is functionally involved in CD83 expression and IL-12 induction. Eur. J. Immunol..

[24] Glaria E., Valledor A.F. (2020). Roles of CD38 in the Immune Response to Infection. Cells.

[25] Ogiya D., Liu J., Ohguchi H., Kurata K., Samur M.K., Tai Y.T., Adamia S., Ando K., Hideshima T., Anderson K.C. (2020). The JAK-STAT pathway regulates CD38 on myeloma cells in the bone marrow microenvironment: Therapeutic implications. Blood.

[26] Qiu Y., Xu S., Chen X., Wu X., Zhou Z., Zhang J., Tu Q., Dong B., Liu Z., He J. (2023). NAD(+) exhaustion by CD38 upregulation contributes to blood pressure elevation and vascular damage in hypertension. Signal. Transduct. Target. Ther..

[27] McReynolds M.R., Chellappa K., Baur J.A. (2020). Age-related NAD(+) decline. Exp. Gerontol..

[28] Hogan K.A., Chini C.C.S., Chini E.N. (2019). The Multi-faceted Ecto-enzyme CD38: Roles in Immunomodulation, Cancer, Aging, and Metabolic Diseases. Front. Immunol..

[29] Gan L., Liu D., Liu J., Chen E., Chen C., Liu L., Hu H., Guan X., Ma W., Zhang Y. (2021). CD38 deficiency alleviates Ang II-induced vascular remodeling by inhibiting small extracellular vesicle-mediated vascular smooth muscle cell senescence in mice. Signal. Transduct. Target. Ther..

[30] Itani H.A., Xiao L., Saleh M.A., Wu J., Pilkinton M.A., Dale B.L., Barbaro N.R., Foss J.D., Kirabo A., Montaniel K.R. (2016). CD70 Exacerbates Blood Pressure Elevation and Renal Damage in Response to Repeated Hypertensive Stimuli. Circ. Res..

[31] Lopez Gelston C.A., Balasubbramanian D., Abouelkheir G.R., Lopez A.H., Hudson K.R., Johnson E.R., Muthuchamy M., Mitchell B.M., Rutkowski J.M. (2018). Enhancing Renal Lymphatic Expansion Prevents Hypertension in Mice. Circ. Res..

[32] Navaneethabalakrishnan S., Goodlett B.L., Smith H.L., Cardenas A., Burns A., Mitchell B.M. (2024). Differential changes in end organ immune cells and inflammation in salt-sensitive hypertension: Effects of lowering blood pressure. Clin. Sci..

[33] Navaneethabalakrishnan S., Goodlett B.L., Smith H.L., Montalvo R.A., Cardenas A., Mitchell B.M. (2024). Differential changes in end organ immune cells and inflammation in salt-sensitive hypertension: Effects of increasing M2 macrophages. Clin. Sci..

[34] Goodlett B.L., Kang C.S., Yoo E., Navaneethabalakrishnan S., Balasubbramanian D., Love S.E., Sims B.M., Avilez D.L., Tate W., Chavez D.R. (2021). A Kidney-Targeted Nanoparticle to Augment Renal Lymphatic Density Decreases Blood Pressure in Hypertensive Mice. Pharmaceutics.

[35] Amend S.R., Valkenburg K.C., Pienta K.J. (2016). Murine Hind Limb Long Bone Dissection and Bone Marrow Isolation. J. Vis. Exp..

[36] Assouvie A., Daley-Bauer L.P., Rousselet G. (2018). Growing Murine Bone Marrow-Derived Macrophages. Methods Mol. Biol..

[37] Na Y.R., Jung D., Gu G.J., Seok S.H. (2016). GM-CSF Grown Bone Marrow Derived Cells Are Composed of Phenotypically Different Dendritic Cells and Macrophages. Mol. Cells..

[38] Toda G., Yamauchi T., Kadowaki T., Ueki K. (2021). Preparation and culture of bone marrow-derived macrophages from mice for functional analysis. STAR Protoc..

[39] Sauter M., Sauter R.J., Nording H., Olbrich M., Emschermann F., Langer H.F. (2022). Protocol to isolate and analyze mouse bone marrow derived dendritic cells (BMDC). STAR Protoc..

[40] Barbaro N.R., Foss J.D., Kryshtal D.O., Tsyba N., Kumaresan S., Xiao L., Mernaugh R.L., Itani H.A., Loperena R., Chen W. (2017). Dendritic Cell Amiloride-Sensitive Channels Mediate Sodium-Induced Inflammation and Hypertension. Cell Rep..

[41] Dong M.B., Rahman M.J., Tarbell K.V. (2016). Flow cytometric gating for spleen monocyte and DC subsets: Differences in auto-immune NOD mice and with acute inflammation. J. Immunol. Methods..

[42] Lee K.M.C., Achuthan A.A., Hamilton J.A. (2020). GM-CSF: A Promising Target in Inflammation and Autoimmunity. Immunotargets Ther..

[43] Filippatos G.S., Kardaras F. (2002). Chemokines and other novel inflammatory markers in hypertension: What can their plasma levels tell us?. Int. J. Cardiol..

[44] Parissis J.T., Korovesis S., Giazitzoglou E., Kalivas P., Katritsis D. (2002). Plasma profiles of peripheral monocyte-related inflammatory markers in patients with arterial hypertension. Correlations with plasma endothelin-1. Int. J. Cardiol..

[45] Parissis J.T., Venetsanou K.F., Kalantzi M.V., Mentzikof D.D., Karas S.M. (2000). Serum profiles of granulocyte-macrophage col-ony-stimulating factor and C-C chemokines in hypertensive patients with or without significant hyperlipidemia. Am. J. Cardiol..

[46] Achuthan A., Aslam A.S.M., Nguyen Q., Lam P.Y., Fleetwood A.J., Frye A.T., Louis C., Lee M.C., Smith J.E., Cook A.D. (2018). Glucocorticoids promote apoptosis of proinflammatory monocytes by inhibiting ERK activity. Cell Death Dis..

[47] Hansen G., Hercus T.R., McClure B.J., Stomski F.C., Dottore M., Powell J., Ramshaw H., Woodcock J.M., Xu Y., Guthridge M. (2008). The structure of the GM-CSF receptor complex reveals a distinct mode of cytokine receptor activation. Cell..

[48] van de Laar L., Coffer P.J., Woltman A.M. (2012). Regulation of dendritic cell development by GM-CSF: Molecular control and implications for immune homeostasis and therapy. Blood.

[49] Satou R., Gonzalez-Villalobos R.A. (2012). JAK-STAT and the renin-angiotensin system: The role of the JAK-STAT pathway in blood pressure and intrarenal renin-angiotensin system regulation. JAKSTAT..

[50] Gao H.L., Yu X.J., Feng Y.Q., Yang Y., Hu H.B., Zhao Y.Y., Zhang J.H., Liu K.L., Zhang Y., Fu L.Y. (2023). Luteolin At-tenuates Hypertension via Inhibiting NF-kappaB-Mediated Inflammation and PI3K/Akt Signaling Pathway in the Hypotha-lamic Paraventricular Nucleus. Nutrients.

[51] Ikezumi Y., Hurst L.A., Masaki T., Atkins R.C., Nikolic-Paterson D.J. (2003). Adoptive transfer studies demonstrate that macrophages can induce proteinuria and mesangial cell proliferation. Kidney Int..

[52] Li W., Li Y., Jin X., Liao Q., Chen Z., Peng H., Zhou Y. (2022). CD38: A Significant Regulator of Macrophage Function. Front. Oncol..

[53] Jablonski K.A., Amici S.A., Webb L.M., Ruiz-Rosado Jde D., Popovich P.G., Partida-Sanchez S., Guerau-de-Arellano M. (2015). Novel Markers to Delineate Murine M1 and M2 Macrophages. PLoS ONE..

[54] Guan X.H., Hong X., Zhao N., Liu X.H., Xiao Y.F., Chen T.T., Deng L.B., Wang X.L., Wang J.B., Ji G.J. (2017). CD38 promotes angiotensin II-induced cardiac hypertrophy. J. Cell Mol. Med..

[55] Xiao L., Kirabo A., Wu J., Saleh M.A., Zhu L., Wang F., Takahashi T., Loperena R., Foss J.D., Mernaugh R.L. (2015). Renal De-nervation Prevents Immune Cell Activation and Renal Inflammation in Angiotensin II-Induced Hypertension. Circ. Res..

[56] Huang L., Wang A., Hao Y., Li W., Liu C., Yang Z., Zheng F., Zhou M.S. (2018). Macrophage Depletion Lowered Blood Pressure and Attenuated Hypertensive Renal Injury and Fibrosis. Front. Physiol..

[57] De Ciuceis C., Amiri F., Brassard P., Endemann D.H., Touyz R.M., Schiffrin E.L. (2005). Reduced vascular remodeling, endothelial dysfunction, and oxidative stress in resistance arteries of angiotensin II-infused macrophage colony-stimulating factor-deficient mice: Evidence for a role in inflammation in angiotensin-induced vascular injury. Arter. Thromb. Vasc. Biol..

[58] Srinivas B., Alluri K., Rhaleb N.E., Belmadani S., Matrougui K. (2024). Role of plasmacytoid dendritic cells in vascular dysfunction in mice with renovascular hypertension. Heliyon.

[59] Dudziak D., Kamphorst A.O., Heidkamp G.F., Buchholz V.R., Trumpfheller C., Yamazaki S., Cheong C., Liu K., Lee H.W., Park C.G. (2007). Differential antigen processing by dendritic cell subsets in vivo. Science.

[60] Lewis K.L., Caton M.L., Bogunovic M., Greter M., Grajkowska L.T., Ng D., Klinakis A., Charo I.F., Jung S., Gommerman J.L. (2011). Notch2 receptor signaling controls functional differentiation of dendritic cells in the spleen and intestine. Immunity.

[61] Merad M., Sathe P., Helft J., Miller J., Mortha A. (2013). The dendritic cell lineage: Ontogeny and function of dendritic cells and their subsets in the steady state and the inflamed setting. Annu. Rev. Immunol..

[62] Lewis K.L., Reizis B. (2012). Dendritic cells: Arbiters of immunity and immunological tolerance. Cold Spring Harb. Perspect. Biol..

[63] Kasahara S., Clark E.A. (2012). Dendritic cell-associated lectin 2 (DCAL2) defines a distinct CD8alpha- dendritic cell subset. J. Leukoc. Biol..

