# Supplementary material for: Elevated Salt or Angiotensin II Levels Induce CD38+ Innate Immune Cells in the Presence of Granulocyte-Macrophage Colony Stimulating Factor
Source: Cells. 2024 Aug 4;13(15):1302. doi: 10.3390/cells13151302 (PMC11311366; doi:10.3390/cells13151302)
Supplement: Supplementary file 1 [file cells-13-01302-s001.zip › CD38-Cells-2-Supplement.pdf]

Panel	Fortessa Flow Cytometry Antibody Panel						
Fluorochrome	APC	APC-Cy7	BV421	BV785	FITC	PE-Cy7	Ghost Dye 710
Antigen	CD11c	CD38	CD45.2	CD206	CD11b	F4/80	LIVE/DEAD
Dilution Factor	1:100 KIDNEYS AND 1:200 CULTURED CELLS						1:400
Clone	N418	90	104	C068C2	M1/70	BM8	
Manufacturer	BioLegend	BioLegend	BD	BioLegend	BioLegend	BloLegend	Tonbo

(**a**)

Panel	Cytek Flow Cytometry Antibody Panel												
Fluorochrome	APC	APC-Cy7	BUV395	BUV496	BUV737	BV421	BV711	BV785	BV605	BV650	FITC	PerCP-eFluor ™ 710	PE-Cy7	Zombie UV
Antigen	CD11c	CD38	Siglec-H	CD86	Ly-6C	CD45.2	CD68	CD206	XCIR1	MHCII	CD11b	DCIR2	F4/80	LIVE/DEAD
Dilution Factor	1:100 KIDNEYS AND 1:200 CULTURED CELLS													1:200
Clone	N418	90	551	PO3	HK1.4rMAb	104	FA-11	C068C2	ZET	M5/114.15.2	M1/70	33D1	BM8	
Manufacturer	BioLegend	BioLegend	BD Biosciences	BD Biosciences	BD Biosciences	BD	BioLegend	BioLegend	BioLegend	BioLegend	BioLegend	Thermo Fisher Scientific	BloLegend	BioLegend

(**b**)

Panel	Adoptive Transfer Flow Cytometry Antibody Panel							
Fluorochrome	APC	APC-Cy7	BV421	BV785	FITC	PE-Cy7	CellTracker™ Deep Red Dye	Zombie UV
Antigen	CD11c	CD38	CD45.2	CD206	CD11b	F4/80		LIVE/DEAD
Dilution Factor	1:100 KIDNEYS AND 1:200 CULTURED CELLS							1:200
Clone	N418	90	104	C068C2	M1/70	BM8		
Manufacturer	BioLegend	BioLegend	BD	BioLegend	BioLegend	BloLegend	Thermo Fisher Scientific	BioLegend

(**c**)

Supplementary Figure S1. Antibody panels for flow cytometry. (**a**) Fortessa flow cytometer panel was used to identify macrophage and DC subtypes *in vivo* and *in vitro* on the Fortessa instrument; (**b**) Cytek flow cytometry antibody panel was used to identify macrophage and DC subtypes *in vivo* and *in vitro* on the Cytek instrument; (**c**) adoptive transfer flow cytometry antibody panel was used on the Cytek and to identify the cells that were adoptively transferred into the hypertensive mice.

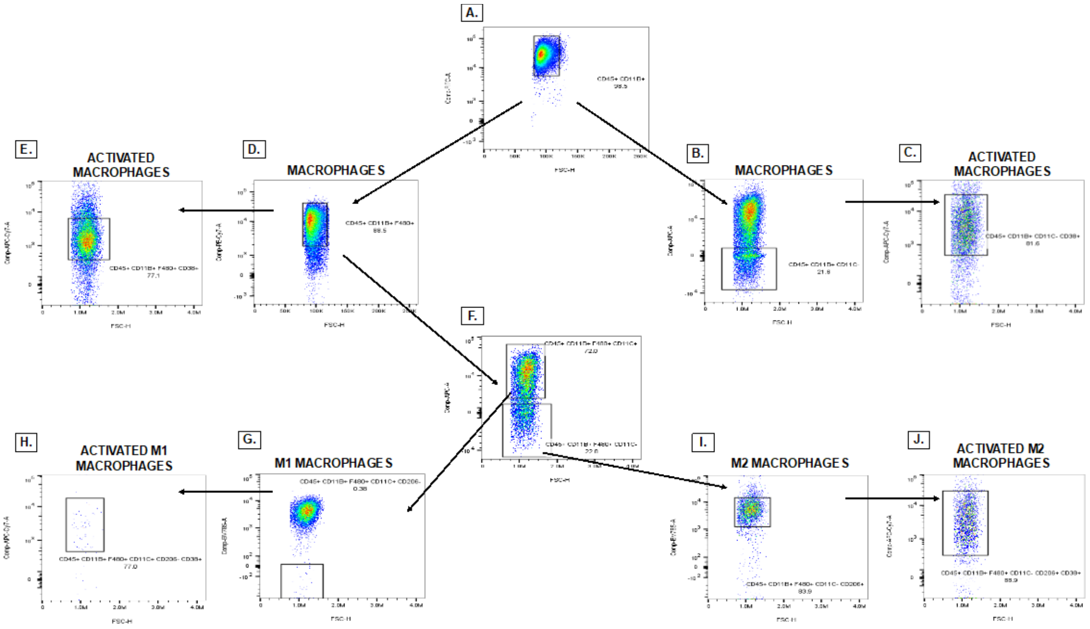

(a)

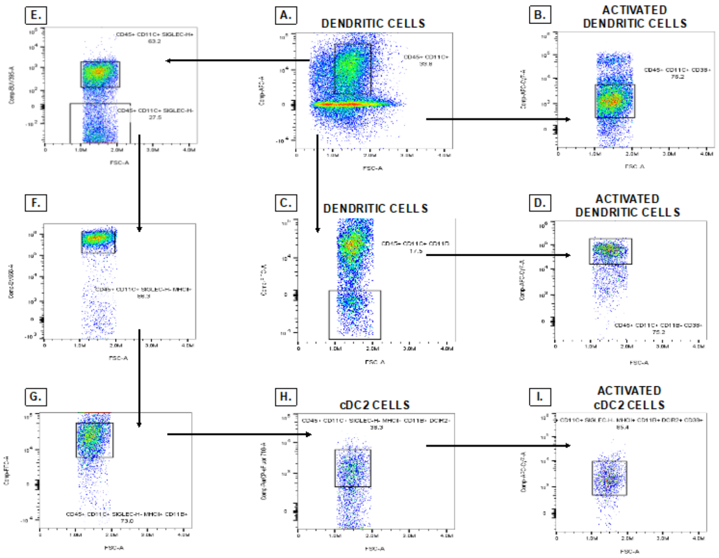

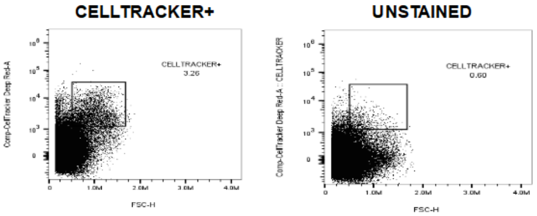

(c)

(b)

Supplementary Figure S2. Flow cytometry gating strategies for innate immune cells. All flow cytometry panels were initially gated with a live/dead gate, single cell gate, and CD45+ gate before proceeding to the first gate of each strategy. For the macrophage gating strategy (**a**) starting at gate A (CD45+ CD11b+), gates for CD11c- (B) or F480+ (D) macrophages can be added to differentiate the two macrophage phenotypes. The progression of A to B to C shows how to gate for CD11c- macrophages as well as their CD38+ activated phenotype (C). The progression of A to D to E shows the gating strategy for F480+ macrophages as well as their CD38+ activated phenotype (E). To gate for M1 or M2 macrophages, gate D proceeds to gate F where M1 macrophages can be gated from the CD11c + gate in gate F and M2 macrophages can be gated from the CD11c- gate in gate F. Gate G shows the full M1 gating strategy, with gate H showing CD38+ activated M1 Macrophages. Proceeding from the CD11c- population in gate F, M2 macrophages are fully gated in gate I with their activated CD38+ phenotype gated for in gate J; for the DC gating strategy (**b**) starting at gate A (CD45+ CD11c+), moving to gate B this is CD38+ activated CD45+ CD11c+ DCs. Progressing from gate A to gate C shows another DC gating strategy where they are CD11b- and in gate D these cells are gated for their CD38+

activated phenotype. Stating at gate A again, we can now proceed with gating for cDC2s. Moving from gate A to E to F to G to H, we get the full gating strategy for cDC2s. Gate I is the CD38+ activated cDc2s. (**c**) Gating strategy for adoptive transfer Celltracker+ cells.

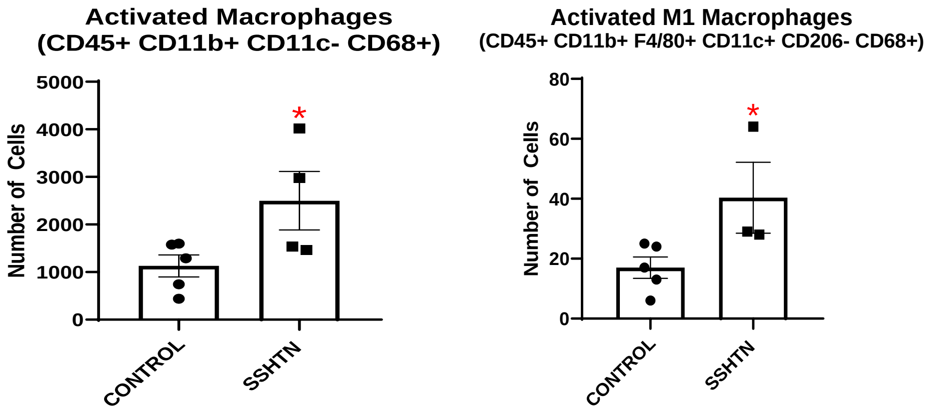

(**a**)

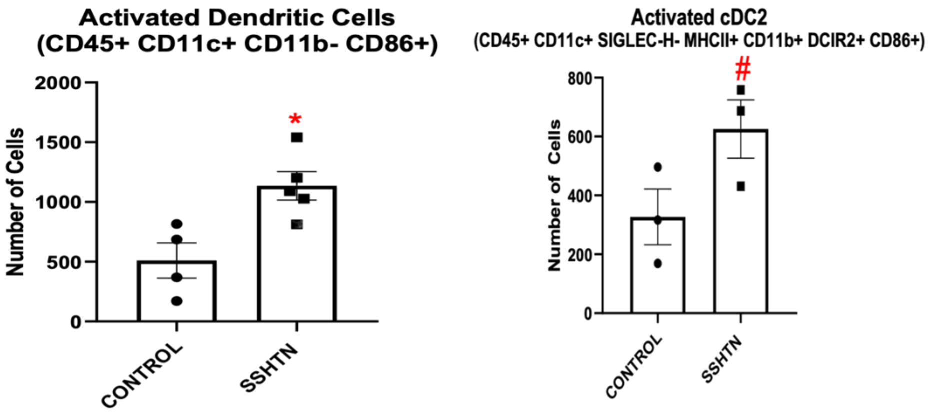

(**b**)

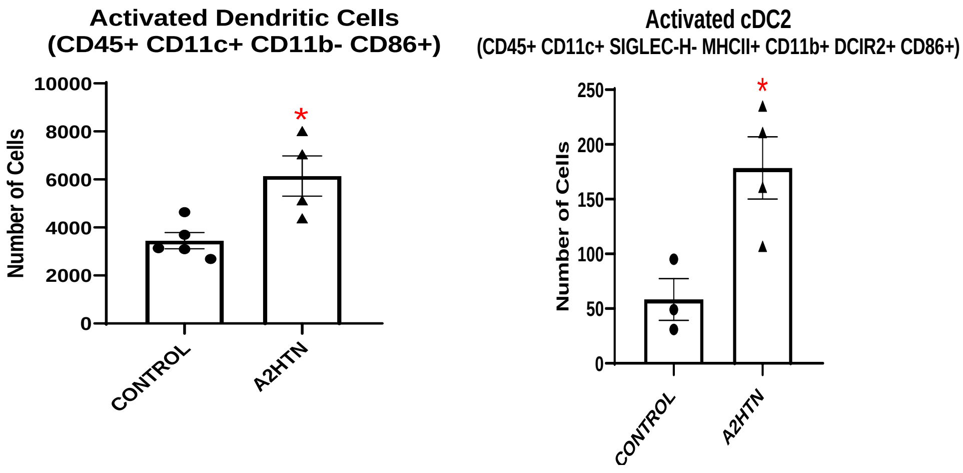

(**c**)

Supplementary Figure S3. Activated macrophages and M1 macrophages are increased in SSHTN and DCs and cDC2s are increased in SSHTN and A2HTN. Populations of (**a**) CD68+ macrophages and (**b**) CD86+ DCs in SSHTN and (**c**) CD86+ DCs in A2HTN.

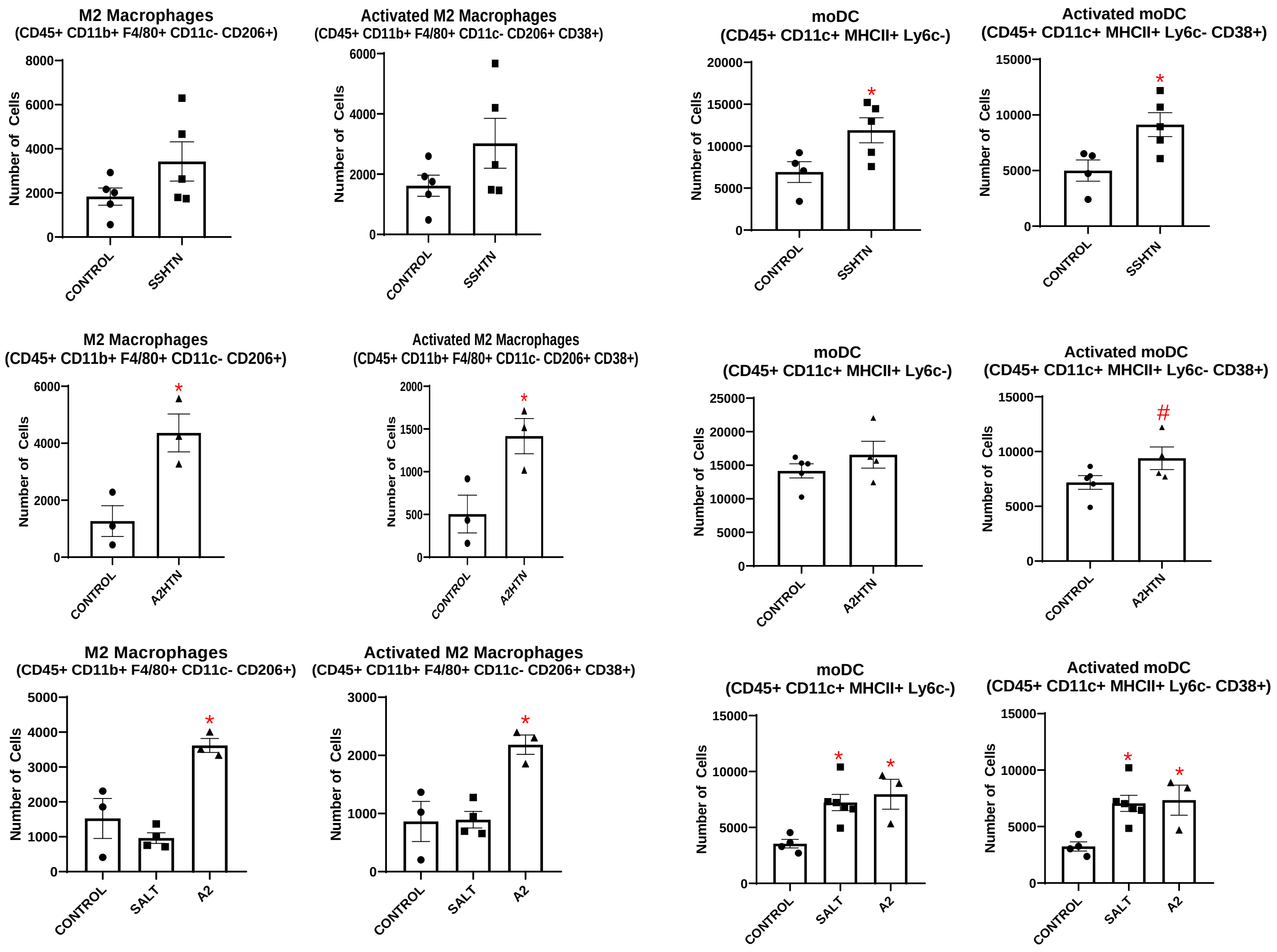

(a)

(b)

Supplementary Figure S4. SSHTN and A2HTN *in vivo* and salt and A2 treatment *in vitro* differentially affect innate immune cell populations. Populations of (**a**) M2 macrophages, (**b**) moDCs, (**c**) cDC1s, and (**d**) alternatively gated cDC2s in SSHTN, A2HTN, and *in vitro* models.

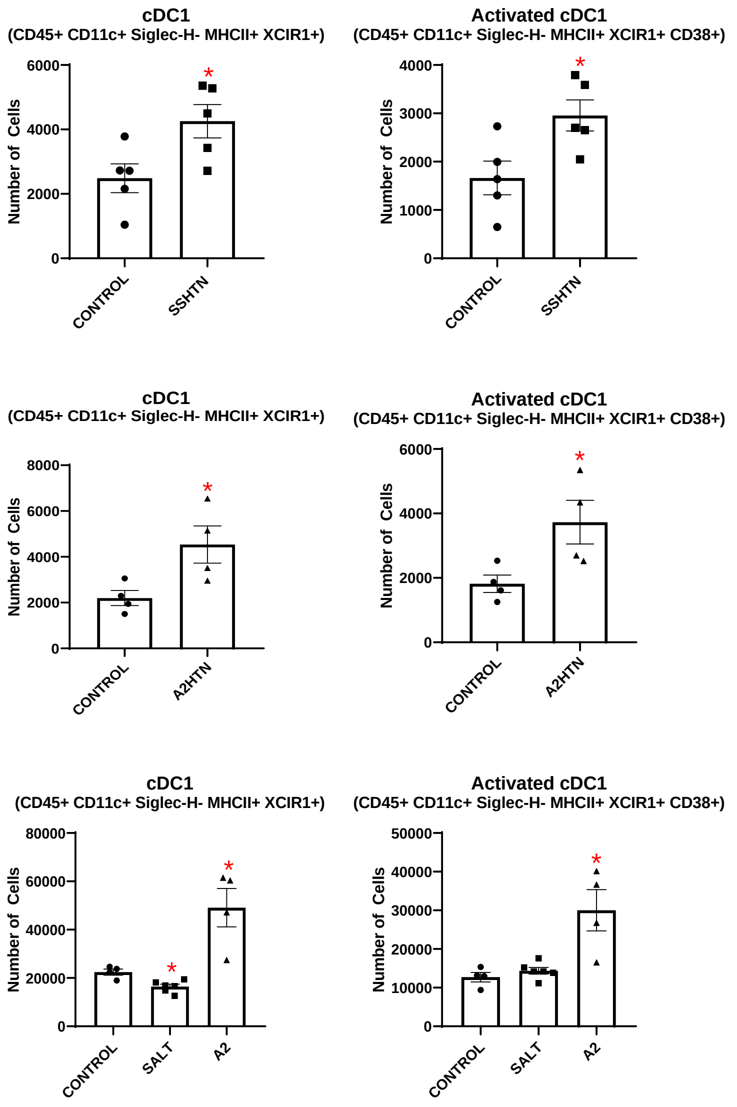

(c)

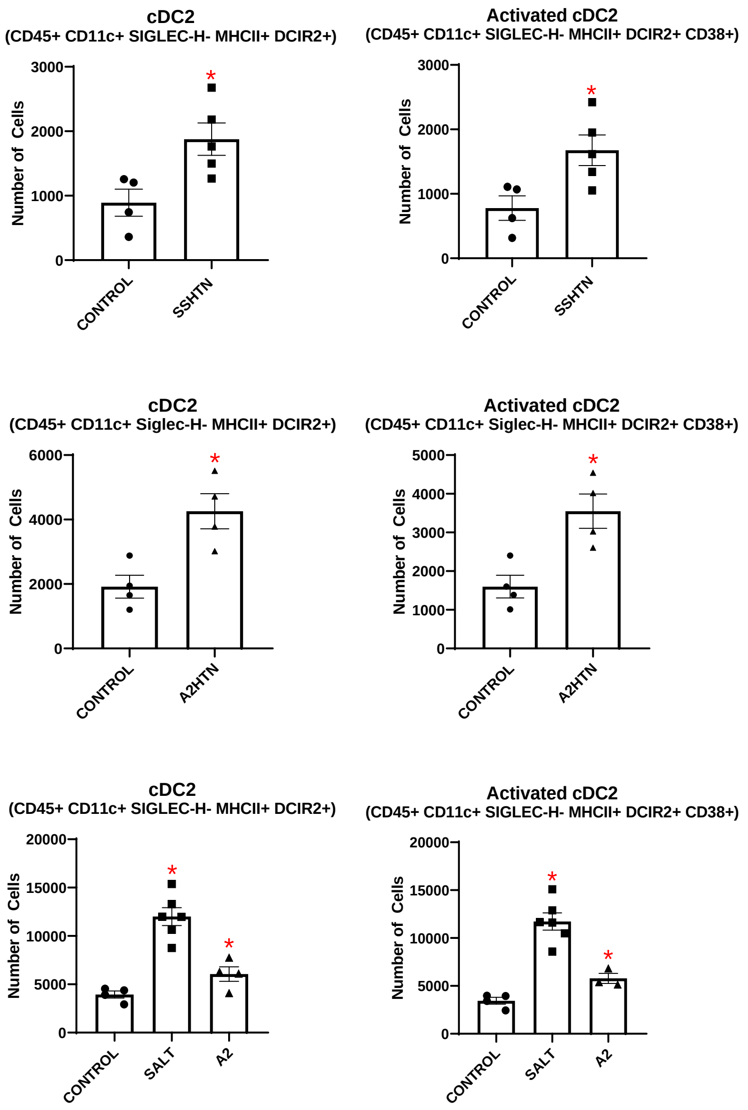

(d)

Supplementary Figure S4 cont.

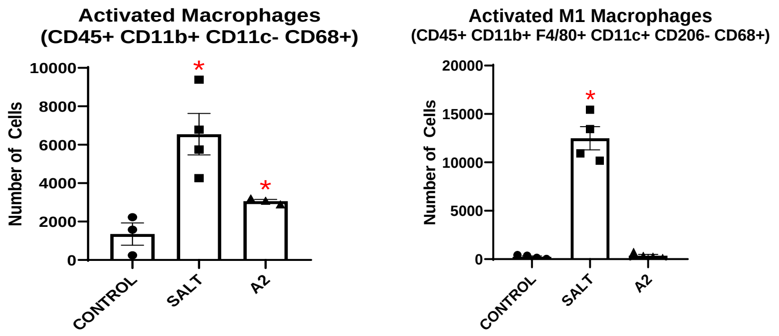

(a)

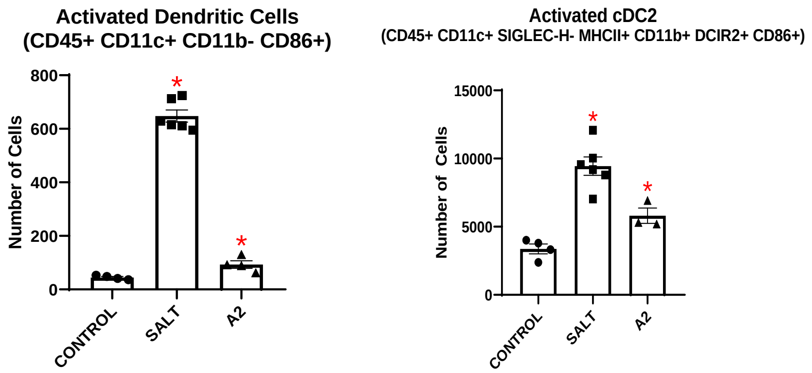

(b)

Supplementary Figure S5. Treatment of BMDMs with hypertensive stimuli increase activated macrophages, M1 macrophages, DCs, and cDC2s. Populations of (**a**) CD68+ macrophages and (**b**) CD86+ DCs following treatment of BMDMs with salt or A2.
